# Pacemaking Property of RVLM Presympathetic Neurons

**DOI:** 10.3389/fphys.2016.00424

**Published:** 2016-09-22

**Authors:** Daniela Accorsi-Mendonça, Melina P. da Silva, George M. P. R. Souza, Ludmila Lima-Silveira, Marlusa Karlen-Amarante, Mateus R. Amorim, Carlos E. L. Almado, Davi J. A. Moraes, Benedito H. Machado

**Affiliations:** Department of Physiology, School of Medicine of Ribeirão Preto, University of São PauloSão Paulo, Brazil

**Keywords:** neurogenic hypertension, sympathetic activity, presympathetic neurons

## Abstract

Despite several studies describing the electrophysiological properties of RVLM presympathetic neurons, there is no consensus in the literature about their pacemaking property, mainly due to different experimental approaches used for recordings of neuronal intrinsic properties. In this review we are presenting a historical retrospective about the pioneering studies and their controversies on the intrinsic electrophysiological property of auto-depolarization of these cells in conjunction with recent studies from our laboratory documenting that RVLM presympathetic neurons present pacemaking capacity. We also discuss whether increased sympathetic activity observed in animal models of neurogenic hypertension (CIH and SHR) are dependent on changes in the intrinsic electrophysiological properties of these cells or due to changes in modulatory inputs from neurons of the respiratory network. We also highlight the key role of I_NaP_ as the major current contributing to the pacemaking property of RVLM presympathetic neurons.

## RVLM and sympathetic outflow

Presympathetic neurons located in the rostral ventrolateral medulla (RVLM) are responsible for generating the sympathetic drive to the cardiovascular system and ultimately determine cardiac output and vascular resistance (Dampney, 1994). Original studies by Owsjannikow and Dittmar, from Carl Ludwig's laboratory, suggested the presence of a vasomotor center in the medulla (apud Seller, 1996). These authors performed controlled lesions in the brainstem and simultaneously recorded arterial pressure. After several precise anteroposterior transections in the brain axis, they observed that a small area in the ventrolateral medulla was critical to keep the baseline arterial pressure and identified this region as a vasomotor area (apud Seller, 1996). These findings by the Germans physiologists, in the second half of the 19th century, were the first description of the pressor area and contributed to the identification of spinally projecting sympatho-excitatory neurons.

Additional evidence about the relevance of RVLM in the maintenance of baseline arterial pressure was provided in a study by Guertzenstein and Silver (1974), in which they demonstrated that bilateral inhibition of specific areas in the ventral medulla, using inhibitory amino acid glycine, produced a large fall in the arterial blood pressure, similar to that described by Dittmar after medullo-spinal transections. The role of RVLM in controlling the cardiovascular function was also described in a study by Granata et al. (1983), which reinforced the concept of a key region in the medullary surface for the maintenance of arterial blood pressure. Moreover, RVLM activation by either electrical stimulation or application of excitatory amino acid (glutamate) or even RVLM disinhibition by application of GABA receptor antagonist (bicuculline), in anesthetized or conscious animals, elicited an increase in sympathetic activity and arterial blood pressure (Willette et al., 1983; Reis et al., 1984; Ross et al., 1984a; de Paula and Machado, 2000; Sakima et al., 2000; Moraes et al., 2011), while bilateral electrolytic lesions, microinjection of GABA or administration of tetrodotoxin, leads to a large fall in the arterial pressure to levels comparable to those observed after transection below brainstem (Dampney and Moon, 1980; Willette et al., 1983; Reis et al., 1984; Benarroch et al., 1986).

Fine anatomical studies by Amendt et al. (1979) and Ross et al. (1981, 1984a) using retrograde transport and immunocytochemical technique demonstrated that RVLM neurons project directly to the thoracic spinal cord, where preganglionic sympathetic neurons are located. Studies by Ross et al. (1984a,b) also demonstrated that terminals of RVLM neurons contain phenylethanolamine-N-methyl transferase (PNMT), characterizing these neurons as C1 adrenaline-synthesizing neurons. Photostimulation of this neuronal phenotype, using lentivirus that expresses channelrhodopsin-2, increased the sympathetic nerve activity and arterial blood pressure in rats *in vivo* confirming the involvement of these cells in the cardiovascular regulation (Abbott et al., 2009).

The direct neural projection from RVLM neurons to spinal cord was also electrophysiologically characterized using antidromic stimulation (Barman and Gebber, 1985; McAllen, 1986; Morrison et al., 1988). In addition to their spinally projection, RVLM neurons are also characterized by the reduction in their firing frequency or silence completely in face of baroreflex stimulation (Schreihofer and Guyenet, 1997). Therefore, it is very well documented that there are presympathetic neurons in RVLM and that their integrity is essential to maintain the level of sympathetic activity and, consequently, the baseline levels of arterial blood pressure.

## Electrophysiological characterization of presympathetic neurons

After anatomical and functional evidence that cell bodies of presympathetic neurons were located at RVLM (Amendt et al., 1979; Ross et al., 1981, 1984a; Barman and Gebber, 1985; Morrison et al., 1988) several studies were performed to evaluate their pacemaker activity. Initially, studies using anesthetized animals described that RVLM neurons presented tonic activity, a state of continuous discharge of action potential, and their firing was highly synchronized with the sympathetic nerve discharge, the arterial pulse and respiration (Barman and Gebber, 1985; Haselton and Guyenet, 1989; Granata and Kitai, 1992). There is also experimental evidence that the firing frequency of RVLM presympathetic neurons is modulated by the afferents inputs from the arterial baroreceptors (Barman and Gebber, 1985; McAllen, 1986; Granata and Kitai, 1992).

A very important study by Sun et al. (1988a) considered two theories to explain the tonic activity of RVLM presympathetic neurons observed in anesthetized animals: (1) the *pacemaker theory*, suggesting that these neurons have intrinsic capacity to generate rhythmic activity and (2) the *network theory* suggesting that the activity of these neurons is dependent on the balance of tonic excitatory and inhibitory synaptic inputs arising from other brain regions. Although, different studies have documented the presence of excitatory and inhibitory inputs to RVLM neurons (Brown and Guyenet, 1985; Cravo and Morrison, 1993; Dampney, 1994; Schreihofer et al., 2000; Schreihofer and Guyenet, 2002; Gao and Derbenev, 2013), the main issue about these cells was related to their pacemaking capacity. More recently, it was documented that glial cells are also involved in the control of arterial pressure, since selective stimulation of RVLM astrocytes, using optogenetic approach, induced ATP release, depolarization of the presympathetic neurons with consequent increase in the sympathetic nerve activity and arterial pressure (Marina et al., 2013). Therefore, the controversy about the capacity the RVLM neurons generate spontaneous and rhythmic activity persisted by several years.

In this context, Sun et al. (1988a) provided evidence supporting the concept that RVLM neurons, under experimental conditions in which synaptic activity is low, are pacemakers. These authors using anesthetized adult rats and *in vitro* experiments (bloc of vascularly perfused bulb), reduced the excitatory neurotransmission using glutamate-receptor antagonist (kynurenic acid) and recorded the firing frequency of RVLM neurons using extracellular recordings. Intracisternal injection of kynurenic acid increased the firing frequency of functionally identified barosensitive neurons. On the other hand, several studies documented that microinjections of kynurenic acid into RVLM produced no major changes in the sympathetic nerve activity (Sun and Guyenet, 1987; Kiely and Gordon, 1994; Araujo et al., 1999). In a subsequent study, Sun et al. (1988a) suggested that glutamatergic receptor antagonist may also reduce the neuronal activity in CVLM, which sends inhibitory inputs to RVLM neurons, as demonstrated previously by Willette et al. (1984). Studies performed under the effect of kynurenic acid, Sun et al. (1988a) showed that the majority of synaptic inputs to RVLM presympathetic neurons are reduced and that rhythmic firing pattern observed in these cells using extracellular recordings was due to pacemaker activity. Therefore, based on these experiments Sun et al. (1988a) suggested that presympathetic RVLM neurons have intrinsic pacemaker properties. In their study they stated: “The final proof of the pacemaker theory will have to await the result of intracellular recording experiments.”

In order to explore in further detail the possible pacemaker activity of RVLM presympathetic neurons, Sun et al. (1988b) performed intracellular recordings in brainstem slices of young adult rats. They described that RVLM neurons display a typical pacemaker membrane potential trajectory with no evidence of excitatory synaptic inputs. However, in their study it was not used any pharmacological tool to exclude possible inputs from neuronal network. They ruled out neuronal network involvement in the generation of the regular firing frequency of these neurons because no excitatory post-synaptic potentials (EPSPs) were observed in response to intracellular hyperpolarizing currents. Moreover, an important methodological advancement of this study was the use of a dye to identify spinal cord-projecting RVLM neurons, since the RVLM region is functionally, anatomically as well as chemically, heterogeneous. For this purpose, Sun et al. (1988b) performed injection of rhodamine microbeads into the spinal cord (T3 level) in anesthetized animal and few days later they visualized labeled cells on the slices of the ventral medulla. Taking the advantage of retrogradely identified cells and intracellular recordings, Sun et al. (1988b) confirmed that the RVLM presympathetic neurons present electrophysiological properties of auto-depolarization, i.e., characteristics of pacemaker neurons in accordance with the following criteria: (1) pacemaker firing frequency, (2) tonic discharge of at least 4 spikes per second, (3) loss of pacemaker activity during hyperpolarization around −80 mV, (4) absence of detectable EPSPs even during hyperpolarization. However, it is important to mention that the intracellular recordings may damage the neuronal membrane during the penetration of pipette into the cell, producing a leak current, depolarized resting potential (Li et al., 2004) and inactivation of voltage-dependent sodium channels with a consequent decrease in the frequency discharge (Staley et al., 1992).

After these important studies by Sun et al. (1988a,b), several others from different laboratories tried to identify the presence of pacemaker activity in RVLM neurons using different experimental approaches, such as whole-cell patch clamp technique. This approach is more appropriate for recording neuronal activity since it produces less damage to the membrane of recorded cell and provides more information about the intrinsic properties of neurons, such as ionic conductances related to the firing frequency, which is not feasible using extracellular records. In this context, Kangrga and Loewy (1995) performed experiments to analyze the membrane potential of these cells, using brainstem slices from neonatal rats, retrogradely labeled neurons and whole-cell patch clamp. They identified two types of labeled RVLM neurons: pacemaker and non-pacemaker neurons. The pacemaker cells were classified according to the following criteria: the regenerative spontaneous firing frequency at a constant rate and membrane potential trajectory presenting gradual depolarizing interspike ramps. Kangrga and Loewy (1995) also suggested that the intrinsic tonic firing frequency of RVLM neurons was due to the pacemaker activity and not due to the synaptic inputs. However, it is important to note that two factors make the interpretation of these experiments difficult: (1) identification of the intrinsic properties, since no pharmacological antagonism was used to isolate the recorded cell from the neuronal network and, (2) the results obtained using neonatal rats may be different from those observed in adult animals, since the density distribution of ionic channels in neurons is established in the brain development period between P17 and P19 (Beckh et al., 1989; Straka et al., 2005), and the expression and functional properties of several receptors involved in the synaptic transmission may also change during the development (Ben-Ari, 2002; Luján et al., 2005).

Studies by Lipski et al. (1996) also attempted to shed light on the controversy about the pacemaker activity of RVLM neurons and the possible role of the neural network, studying the activity of RVLM neurons using intracellular recordings in anesthetized adult rats. In their work, Lipski et al. (1996) identified RVLM presympathetic neurons by 2 criteria: (1) inhibition of neuronal activity after stimulation of the aortic depressor nerve and (2) antidromic responses evoked by stimulation of RVLM bulbospinal axons. In contrast to the intrinsic pacemaker properties as previously suggested by Sun et al. (1988a,b) and Kangrga and Loewy (1995), the findings by Lipski et al. (1996) indicated that the spontaneous firing frequency in RVLM presympathetic neurons results from synaptic inputs based on the following evidence: (1) action potentials were normally preceded by depolarizing potentials showing features of fast EPSPs and (2) there was no evidence of regular, ramp-like depolarization between action potentials. Therefore, the results by Lipski et al. (1996) raised again new questions about the “pacemaker” activity of RVLM presympathetic neurons and brought for discussion the network theory related to the generation of action potential in RVLM neurons.

In another study, Lipski et al. (1998) evaluated spontaneous firing frequency in acutely dissociated retrogradely labeled RVLM neurons from neonatal rats (13- to 19-days old), in which all cell-to-cell interactions were eliminated. Using whole-cell patch clamp they verified that these cells presented no pacemaker activity and suggested that the depolarization of these neurons observed in the whole animal was dependent on the influence of inhibitory and excitatory tonic projections from different neuronal networks in the brainstem, such as excitatory projections from the nucleus of tractus solitarius to CVLM neurons, which in turn send monosynaptic inhibitory projections to RVLM presympathetic neurons (Agarwal and Calaresu, 1991).

**Figure 1:**
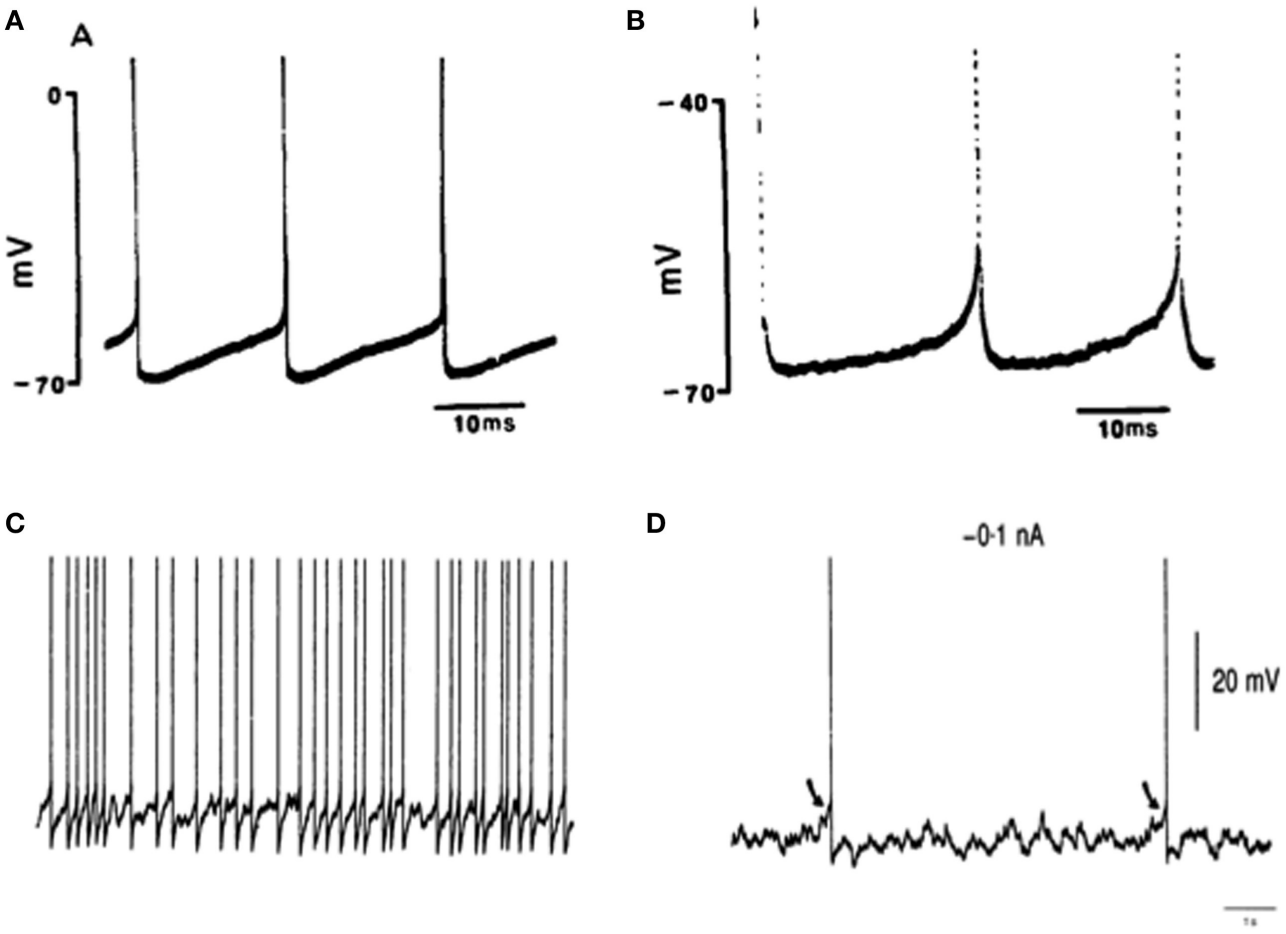
**The activity of RVLM presympathetic neurons**. Typical action potentials of two RVLM neurons **(A,B)** using intracellular recordings in brainstem slices of young adult rats (100–120 g), modified from Sun et al. (1988b, Copyright License Number: 3894201035959). The pacemaker activity of neurons is demonstrated by the resetting after a single spike and absence of detectable EPSPs. **(C,D)** Activity of RVLM neurons using intracellular recordings in anesthetized adult rats (380–500 g), modified from Lipski et al. (1996, Copyright License Number: 3894210262580). Synaptic activity and firing in RVLM neurons (membrane potential: −65 mV, **C**); Synaptic activity and firing during a continuous small polarizing current (−0.1 nA). Arrows indicate EPSPs initiating individual action potentials **(D)**.

Although, the pacemaker property of RVLM presympathetic neurons were evaluated in several studies (Sun et al., 1988a,b; Kangrga and Loewy, 1995; Lipski et al., 1996, 1998) there was no consensus about their spontaneous activity, probably due to different experimental conditions, such as the age of animal, presence of anesthesia and different electrophysiological methods used to recordings the neuronal activity (Figure 1). In addition, it is important to note that the electrophysiological record of RVLM neurons from juvenile and adult rats is not a simple task mainly due to the high degree of technical difficulties in recording neurons in the ventral medulla, a brainstem area presenting high density of myelin.

**Figure 2:**
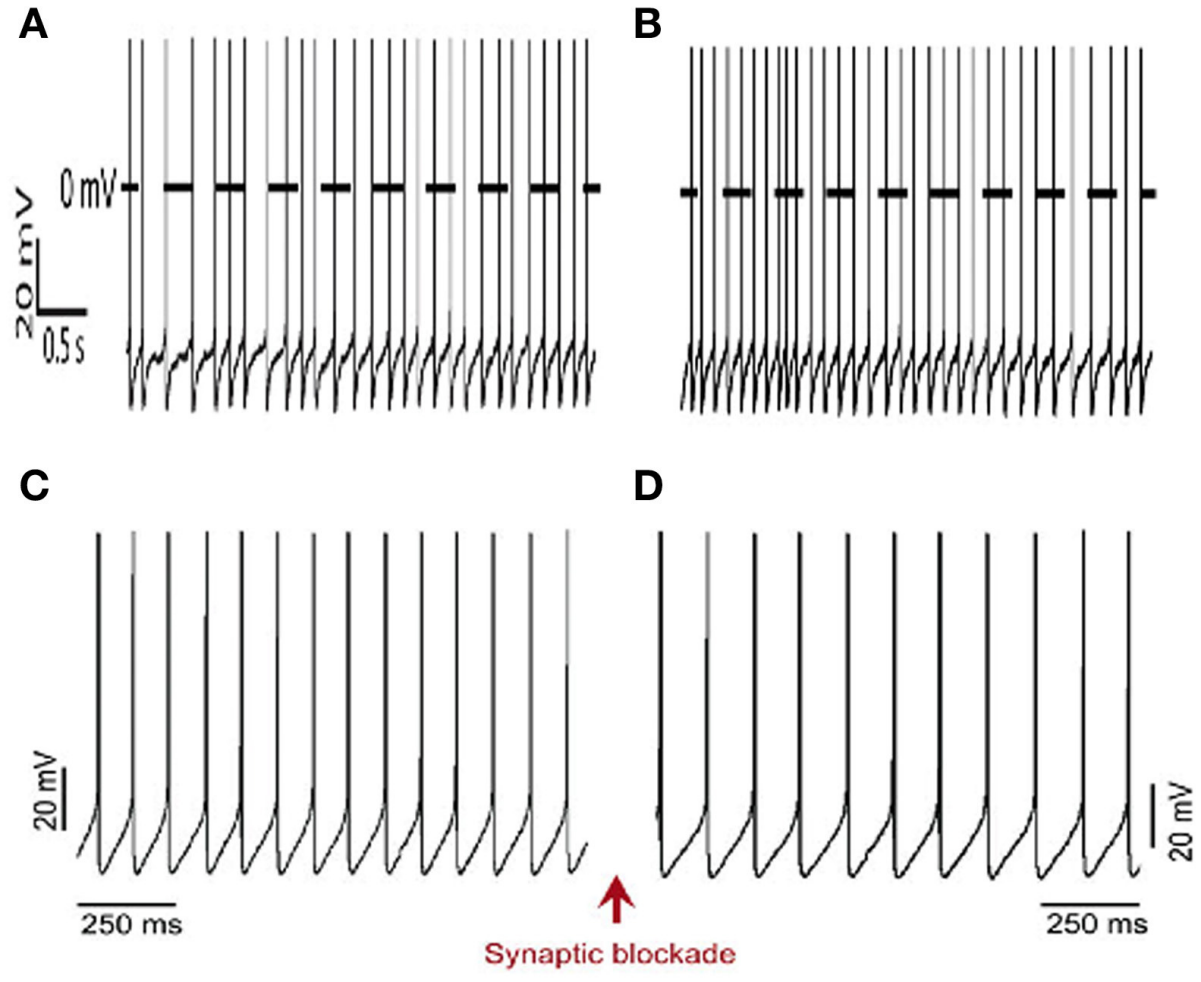
**Pacemaker activity of RVLM neurons. (A)** Spontaneous action potentials of an RVLM neuron using patch clamp technique in *in situ* preparation of juvenile rats (85–140 g) before **(A)** and after blockade **(B)** of fast neurotransmission (2.5–6.0 mM kynurenic acid + 20 μM bicuculline + 1 μM strychnine), modified from Moraes et al. (2013, Copyright order number: 3911900683231). Spontaneous action potentials of an RVLM neuron using patch clamp technique in brainstem slices of juvenile rats (P30–35) before **(C)** and after **(D)** blockade of fast neurotransmission (1 μM strychnine + 20 μM bicuculline + 40 μM AP-5 + 20 μM DNQX), modified from Almado et al. (2014, Copyright License Number: 3894220907506).

In our laboratory, using an *in situ* preparations of juvenile rats (P30-P31), we observed that all RVLM presympathetic neurons fire spontaneously and the frequency of these cells were heterogeneous ranging from 8 to 22 Hz, with depolarized (−52 mV) and hyperpolarized (−63 mV) values of membrane potential (Moraes et al., 2013). Moreover, we observed that respiratory network modulates the activity of RVLM presympathetic neurons, which allow us to classify them into four types, being three of them modulated by respiratory activity. These neurons receive multiple synaptic inputs, observed by a high level of synaptic “noise,” and their baseline firing frequency was probably determined by the balance of excitatory and inhibitory inputs, as observed previously in anesthetized rats by Lipski et al. (1996). However, after pharmacological blockade of fast synaptic transmission in the *in situ* preparation, we clearly verified the intrinsic properties of auto-depolarization in RVLM neurons, confirming their pacemaker properties (Figure 2, Moraes et al., 2013). Due to this discrepancy in the firing rate and resting membrane potential, we combined single cell RT-qPCR and immunohistochemistry to characterize the neurochemical profile of these neurons and we observed that all respiratory-modulated RVLM presympathetic neurons investigated were glutamatergic neurons. However, the expression of tyrosine hydroxylase was detected in the inspiratory-modulated and non-respiratory modulated RVLM presympathetic neurons, but not in the post-inspiratory modulated neurons, pointing out to the existence of different subpopulations of RVLM presympathetic pacemaker neurons (Moraes et al., 2013).

We also performed experiments designed to record retrogradely labeled RVLM presympathetic neurons in brainstem slices preparations from juvenile-adult animals (P35, Almado et al., 2014). Ten days after the surgical procedures to retrogradely label these cells, whole-cell recordings in brainstem slices revealed that RVLM neurons are under synaptic modulation and presented a regular and spontaneous firing frequency, which are in agreement with our findings in the *in situ* preparation. Moreover, after blockade of fast synaptic transmission, the firing frequency of RVLM neurons decreased significantly but their activity was not abolished, indicating that these cells have intrinsic properties required to auto-depolarization. Thus, RVLM presympathetic neurons in slices from juvenile/adults rats also behave as pacemakers under our experimental condition. Therefore, our studies performed in the *in situ* preparation, as well as in the brainstem slices from juvenile-adults rats, support the concept that RVLM presympathetic neurons are indeed pacemakers (Figure 2).

## RVLM neurons and neurogenic hypertension

Several studies have suggested that changes in the intrinsic properties of RVLM neurons are the main cause of cardiovascular disorders, such as neurogenic hypertension, which is characterized by the chronic increase of the arterial blood pressure mediated by sympathetic overactivity rather than vascular and renal dysfunctions (Han et al., 1998; Guyenet, 2006; Toney et al., 2010; Kumagai et al., 2012).

Considering that experimental models of neurogenic hypertension, such as rats submitted to chronic intermittent hypoxia (CIH) and spontaneously hypertensive (SH) rats, show a significant increase in sympathetic tone, more recently we became directly involved with this important issue. Our studies were designed to analyze whether RVLM neurons, from juvenile-adult animals, present an enhancement in their spontaneous firing frequency and whether this enhancement is responsible for the sympathetic overactivity and hypertension observed in CIH and SH rats. To reach these goals, we used *in situ*, as well as *in vitro* preparations. Firstly, we performed blind whole cell patch clamp recordings of RVLM neurons, using *in situ* preparations of juvenile rats (Paton, 1996). This preparation has the advantage of being anesthesia-free with intact brainstem circuits, while the lack of pulsatility makes the brain amenable to whole-cell recordings (Moraes et al., 2013). Although, we have observed an increase in the firing frequency of RVLM presympathetic neurons from CIH rats in the late-expiratory phase of the respiratory cycle (late-E), the blockade of fast synaptic transmission revealed similar intrinsic firing frequency, membrane potential, input resistance as well as intrinsic excitability when compared with RVLM presympathetic neurons from control rats. These important findings show that the sympathetic overactivity observed in this model of neurogenic hypertension is not due to changes in the intrinsic properties of RVLM presympathetic neurons. Therefore, these cells are not in charge of sympathetic overactivity observed in CIH rats. Furthermore, in the intact respiratory and sympathetic brainstem networks, respiratory-modulated RVLM presympathetic neurons from SH rats revealed an increase in their activity, also in the late-expiratory phase, when compared with those neurons from normotensive rats. It is important to highlight that after synaptic blockade, the pacemaking capacity of RVLM presympathetic neurons was similar in either control, CIH or SH rats, indicating clearly that their increased firing frequency during the late-expiratory phase was driven by excitatory synaptic inputs from neurons of the respiratory network (Moraes et al., 2013, 2014).

**Figure 3:**
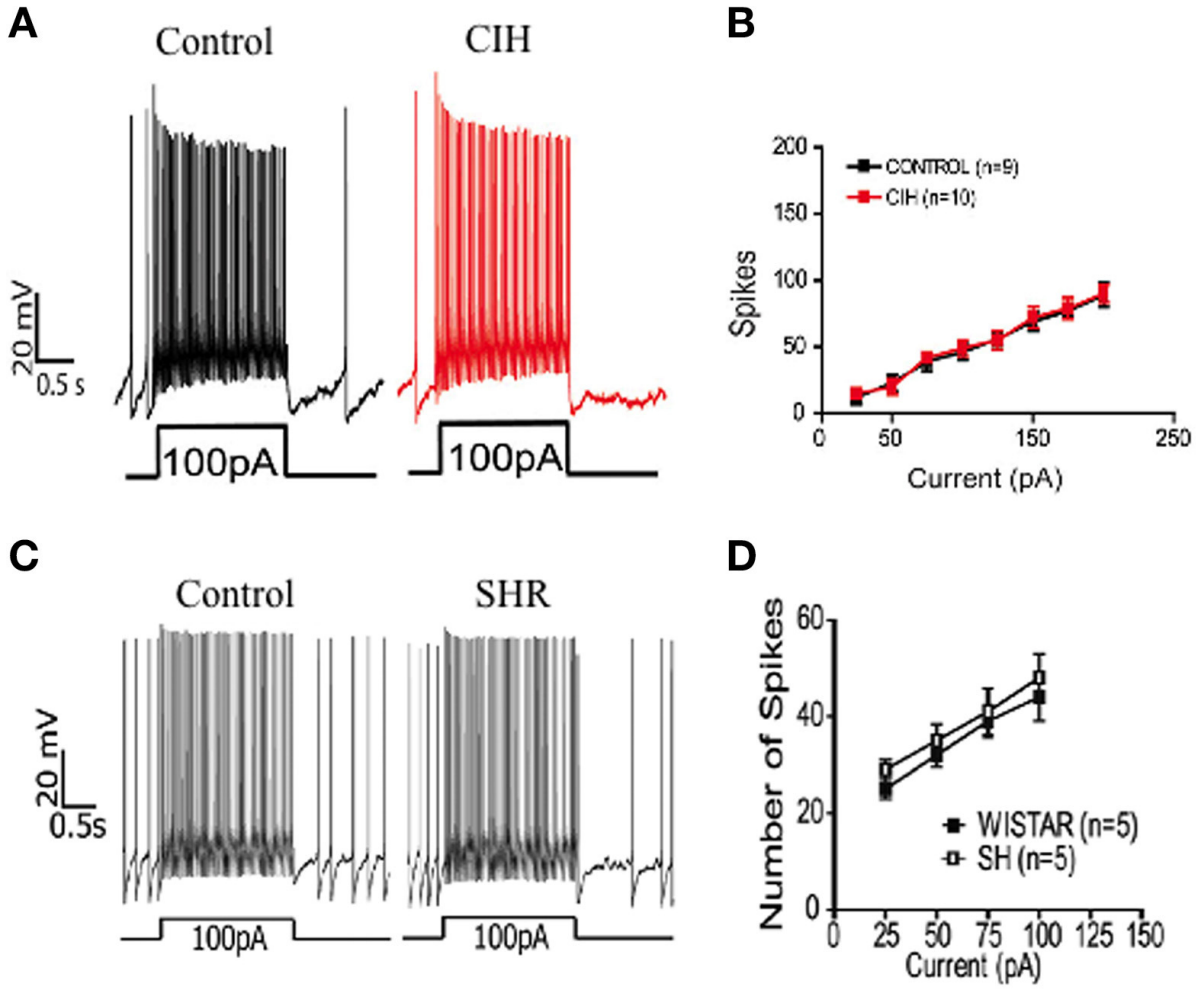
**Intrinsic firing frequency discharge of RVLM neurons in different models of neurogenic hypertension**. Intrinsic firing frequency discharge of RVLM neurons after positive current injection in *in situ* preparation of control and CIH juvenile rats **(A,B)**, modified from Moraes et al. (2013, Copyright order number: 3911900683231); Intrinsic firing frequency discharge of RVLM neurons after positive current injection in *in situ* preparation of control and spontaneous hypertensive rats **(C,D)**, modified from Moraes et al. (2014, Copyright License Number: 3894221294317). All the experiments were performed in the presence of synaptic blockade (2.5–6.0 mM kynurenic acid + 20 μM bicuculline + 1 μM strychnine).

In a series of experiments performed in slices, we also analyzed the effects of CIH on the electrophysiological properties of RVLM presympathetic neurons. As described for the *in situ* approach, in the presence of synaptic blockade RVLM neurons from CIH rats presented no changes in their resting membrane potential, firing frequency and input resistance. Thus, the intrinsic properties of RVLM presympathetic neurons in brainstem slices from juvenile rats exposed to CIH were similar to those observed in neurons from control rats, as we observed in *in situ* preparations of CIH and SH rats. Although, our findings indicate that RVLM presympathetic neurons are pacemakers, our studies using two experimental models of neurogenic hypertension (CIH and SH) also indicate that the increased firing frequency of these cells is not due to changes in their intrinsic properties (Figure 3), but is associated with changes in their modulation by synaptic inputs from the respiratory network.

Taken together, our data, from *in situ* and *in vitro* preparations, allow us to consider that: (1) RVLM presympathetic neurons have intrinsic mechanism that allow them to behave as pacemaker neurons; and (2) the increased respiratory modulation to these neurons is mainly due to excitatory drives (Moraes et al., 2013, 2014), supporting the hypothesis that sympathetic overactivity, present in different models of neurogenic hypertension, might involve changes in the neurons from the respiratory network; the observed changes in these respiratory network neurons seems to increase the excitatory inputs to the RVLM presympathetic neurons (Czyzyk-Krzeska and Trzebski, 1990; Moraes et al., 2013, 2014).

## RVLM presympathetic neurons: conductance and roles in the auto-depolarization

Although, changes in the intrinsic electrophysiological properties of RVLM presympathetic neurons are not the cause of neurogenic hypertension in CIH and SH rats, a considerable amount of effort has been committed to understanding the mechanisms underlying the ability of these neurons to auto-depolarize. Studies by Lipski et al. (1998) using retrograde labeled isolated RVLM neurons demonstrated that these cells express high and low voltage-activated calcium channels. These type of channels presents several functionalities including: (1) neurotransmission, (2) activation of calcium-dependent potassium channels, and (3) neuronal excitability control (Llinás, 1988, 2014; Catterall, 2011). Furthermore, low voltage-activated Ca^2+^ channels have been implicated in the auto-depolarization of RVLM presympathetic neurons during short periods of hypoxia (Sun and Reis, 1994a). However, Kangrga and Loewy (1995), using brainstem slices observed that only in 2 out 13 RVLM presympathetic neurons tested, the application of CdCl_2_, a broad-spectrum calcium channel blocker, abolished the spontaneous firing frequency of cells, while the majority showed a significant enhancement. These results by Kangrga and Loewy (1995) suggested that some RVLM neurons may require Ca^2+^ influx and/or synaptic drive for regenerative firing and are in agreement with studies by Sun and Reis (1994a) demonstrating that increases in the firing frequency of RVLM neurons are synchronized with the rapid increase in Ca^2+^ channel conductance.

In our laboratory, we also investigated the role of calcium channels in the auto-depolarization behavior of RVLM presympathetic neurons. Using *in situ* preparations, we observed that Ni^2+^, a blocker of type T calcium channels, did not eliminate the activity of neurons, but increased their firing frequency (Moraes et al., 2013). However, we believe that type T calcium channels may play an indirect role in the RVLM neurons activity by stimulating calcium-activated potassium channel (BK_Ca_ channels), which in turn may influence the firing frequency of neurons as suggested by Pierrefiche et al. (1995). This suggestion is supported by a slight increase in the action potential duration and the marked decrease in the amplitude and duration of after-hyperpolarization observed previously by Kangrga and Loewy (1995). All together these studies revealed that calcium currents seems to be involved, but are not the main conductance in the intrinsic auto-depolarization observed in the majority of RVLM presympathetic neurons.

A second conductance that seems to be involved in the auto-depolarization of RVLM presympathetic neurons is related to voltage-dependent potassium channels. Previously, it was demonstrated in neonate rats that RVLM presympathetic neurons express a variety of voltage-dependent K^+^ channels (Kangrga and Loewy, 1995; Li et al., 1995). Therefore, this previous information leads us to investigate whether these channels could drive the intrinsic activity of RVLM presympathetic neurons. We documented that RVLM presympathetic neurons show a delay during the depolarizing phase of action potentials generation. In this case, the current that underlies this delayed excitation seems to be similar to transient potassium current. However, when we blocked this conductance using 4-aminopyridine, it resulted in a decrease in after-hyperpolarization amplitude and an increase in the firing frequency. These findings highlight the contribution of this conductance to the action potential kinetics, but not for the auto-depolarization characteristic of RVLM presympathetic neurons (Moraes et al., 2013).

Considering auto-depolarization as a possible summation of a set of smaller conductances operating at membrane potentials just below the spike threshold, we also explored the presence and contribution of such conductances to RVLM presympathetic neurons, mainly those involved in pacemaker activity. The first to be investigated was a current originated by hyperpolarization-activated cyclic-nucleotide-gated channels or HCN channels (McCormick and Pape, 1990; Wahl-Schott and Biel, 2009) that is responsible for keeping the resting potential near to the threshold value. Although, HCN channels strongly modulates spontaneous discharge of several cells (Gu et al., 2005; Rodrigues and Oertel, 2006; Kase and Imoto, 2012), it does not seem to be essential for auto-depolarization of RVLM presympathetic neurons and consequently for blood pressure control, since ZD7288, a specific blocker of these channels, did not change the excitability of RVLM presympathetic neurons, sympathetic activity or mean arterial pressure (Miyawaki et al., 2003; Moraes et al., 2013; Tallapragada et al., 2016).

Another conductance potentially involved in intrinsic auto-depolarization of RVLM neurons is the Na^+^ conductance resistant to TTX, or the I_NaP_ currents. I_NaP_ has been implicated in the regulation of subthreshold excitability in a variety of excitable cells (French and Gage, 1985; Stafstrom et al., 1985). Computational modeling has shown that I_NaP_ is involved with spontaneous excitability due to its contribution for after-hyperpolarization phase increasing the cellular excitability by reducing threshold, and also by increasing the discharge frequency in response to depolarizing current (Vervaeke et al., 2006).

**Figure 4:**
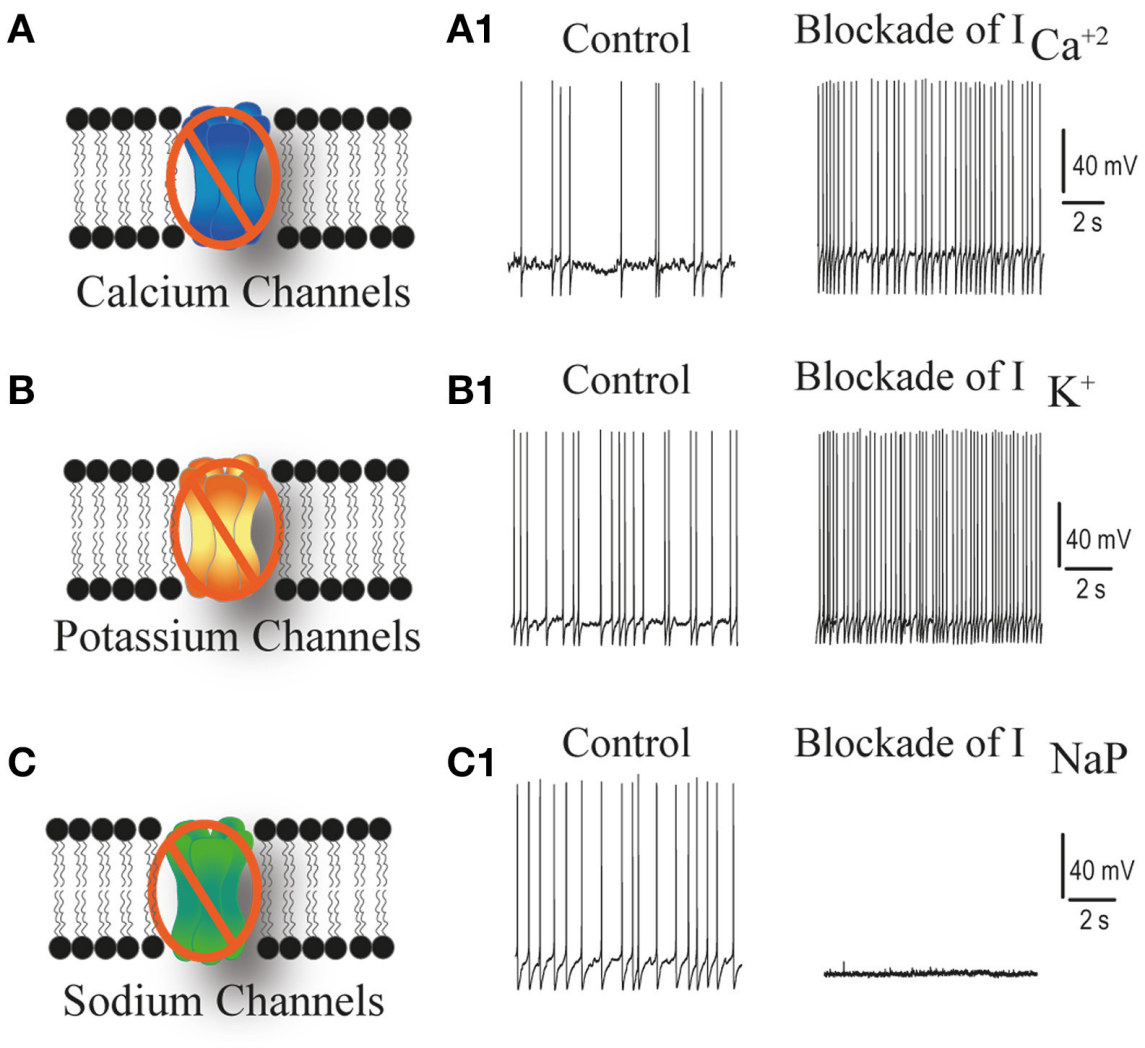
**Schematic representation of the role of RVLM presympathetic neurons conductances in the pacemaker activity**. Schematic representations of calcium, potassium and TTX-resistant sodium channels **(A–C)** and the effect of selective blockade of each channel on intrinsic firing frequency of a pacemaker neuron **(A1–C1)**. Note that blockade of calcium and potassium channels increase the intrinsic firing frequency of RVLM presympathetic neurons while the blockade of TTX-resistant sodium channel abolished the spontaneous activity of these neurons.

Studies by Kangrga and Loewy (1995) demonstrated that the spontaneous firing of RVLM presympathetic neurons is due to a cellular mechanism that is fully dependent on I_NaP_. In a recent study from our laboratory, we obtained similar findings, since a blocker of sodium channels responsible for I_NaP_ (riluzole) abolished the spontaneous activity of these cells (Moraes et al., 2013) revealing the conductance responsible for the intrinsic auto-depolarization of the RVLM presympathetic neurons. Therefore, the I_NaP_ current is essential for the spontaneous activity of these neurons (Figure 4).

Although, our previous studies documented the absence of changes in the intrinsic properties of RVLM presympathetic neurons in neurogenic hypertensive models, we cannot ignore that under some conditions, their intrinsic properties may be altered. There are some studies reporting the possibility that these neurons present chemosensitivity, especially to hypoxia (Sun et al., 1992; Sun and Reis, 1993, 1994a,b; Wang et al., 2001; Koganezawa and Terui, 2007; Koganezawa and Paton, 2014). Therefore, it is possible that RVLM presympathetic neurons present a detection system of brainstem hypoperfusion/ischaemia through specific membrane conductances. It has been emphasized that hypertension can be produced in response to brain hypoperfusion, but the mechanism for detecting this brain condition remains poorly understood (Paton et al., 2009; Cates et al., 2011). We suggest that RVLM presympathetic neurons can switch from synaptically modulated firing frequency to almost pure pacemaking-driven discharge, in a reversible way, during severe hypercapnic/hypoxia, such as gasping and it may represent the last physiological strategic response to increase sympathetic activity and to the survival of the animals under these challenges. It is also important to note that previous studies demonstrated the involvement of intrinsic membrane conductance such as potassium and calcium currents as well as persistent sodium currents for the intrinsic response of RVLM presympathetic neurons exposed to hypoxia (Sun and Reis, 1994a; Koganezawa and Paton, 2014). The contribution of different intrinsic membrane conductances for generating changes in pacemaker RVLM presympathetic neuronal activity in hypoperfusion/ischemia awaits additional experiments and it is a critical step for our understanding of the electrophysiological complexity of the RVLM presympathetic neurons under physiological challenges.

## Conclusion

In this review we described the functional characteristics of RVLM presympathetic neurons and discussed their intrinsic capacity to auto-depolarize and work as pacemakers, a controversial concept in the recent past. Experimental evidence that these neurons are not responsible for sympathetic overactivity observed in models of neurogenic hypertension, such as CIH and SH rats were also discussed. In addition, we highlighted that the main cause of the increased frequency discharge of RVLM presympathetic neurons in these experimental models of neurogenic hypertension is likely related to changes in synaptic inputs from the respiratory network.

## Author contributions

BM, coordinate the group of Ph.D. students, post-docs and young faculty to write this review, wrote several parts of the manuscript and revised the final version; DM, wrote several parts of the manuscript and revised the final version; DA, revised the literature, wrote the manuscript, organized the figures and the revised the final version; MS, revised the literature, wrote the manuscript, organized the figures and the revised the final version; GS, wrote some sections of the manuscript and revised the final version; LS, wrote some sections of the manuscript and revised the final version; MK, wrote some sections of the manuscript and revised the final version; MA, wrote some sections of the manuscript and revised the final version; CA, wrote some sections of the manuscript and revised the final version.

## Funding

The experiments and results from our laboratory presented in this review were part of the Thematic Project funded by FAPESP (2013/06077-5).

### Conflict of interest statement

The authors declare that the research was conducted in the absence of any commercial or financial relationships that could be construed as a potential conflict of interest.
