# Corrigendum: Pacemaking Property of RVLM Presympathetic Neurons

**DOI:** 10.3389/fphys.2016.00575

**Published:** 2016-11-23

**Authors:** Daniela Accorsi-Mendonça, Melina P. da Silva, George M. P. R. Souza, Ludmila Lima-Silveira, Marlusa Karlen-Amarante, Mateus R. Amorim, Carlos E. L. Almado, Davi J. A. Moraes, Benedito H. Machado

**Affiliations:** Department of Physiology, School of Medicine of Ribeirão Preto, University of São PauloSão Paulo, Brazil

**Keywords:** neurogenic hypertension, sympathetic activity, presympathetic neurons

Due to an oversight, the authors did not properly cite two important publications by Roger A. Dampney. In section “RVLM and sympathetic outflow,” the second paragraph should read as follows:

Additional evidence about the relevance of RVLM in the maintenance of baseline arterial pressure was provided in a study by Guertzenstein and Silver (1974), in which they demonstrated that bilateral inhibition of specific areas in the ventral medulla, using inhibitory amino acid glycine, produced a large fall in the arterial blood pressure, similar to that described by Dittmar after medullo-spinal transections. Equally important were the contributions by Dampney (1981) and Dampney et al. (1982), which original studies documented that microinjections of L-glutamate into the ventral medulla increased arterial pressure in anesthetized rabbits. The role of RVLM in controlling the cardiovascular function was also described in a study by Granata et al. (1983), which reinforced the concept of a key region in the medullary surface for the maintenance of arterial blood pressure. Moreover, RVLM activation by either electrical stimulation or application of excitatory amino acid (glutamate) or even RVLM disinhibition by application of GABA receptor antagonist (bicuculline), in anesthetized or conscious animals, elicited an increase in sympathetic activity and arterial blood pressure (Willette et al., 1983; Reis et al., 1984; Ross et al., 1984a; de Paula and Machado, 2000; Sakima et al., 2000; Moraes et al., 2011), while bilateral electrolytic lesions, microinjection of GABA or administration of tetrodotoxin, leads to a large fall in the arterial pressure to levels comparable to those observed after transection below brainstem (Dampney and Moon, 1980; Willette et al., 1983; Reis et al., 1984; Benarroch et al., 1986).

The authors apologize for this oversight. This error does not affect the scientific conclusions of this article in any way.

## Conflict of interest statement

The authors declare that the research was conducted in the absence of any commercial or financial relationships that could be construed as a potential conflict of interest.
